# Agreement between self-report and administrative health data on occurrence of non-cancer chronic disease among participants of the BC generations project

**DOI:** 10.3389/fepid.2022.1054485

**Published:** 2022-12-21

**Authors:** Maryam Darvishian, Jessica Chu, Jonathan Simkin, Ryan Woods, Parveen Bhatti

**Affiliations:** ^1^ Prevention, Screening, and Hereditary Cancer Program, BC Cancer, Vancouver, BC, Canada; ^2^ Cancer Control Research, BC Cancer Research Institute, Vancouver, BC, Canada; ^3^ School of Population and Public Health, Faculty of Medicine, University of British Columbia, Vancouver, BC, Canada; ^4^ Faculty of Health Sciences, Simon Fraser University, Burnaby, BC, Canada

**Keywords:** cohort, self-report, administrative data, chronic disease, concordance

## Abstract

Population-based studies of non-cancer chronic disease often rely on self-reported data for disease diagnosis, which may be incomplete, unreliable and suffer from bias. Recently, the British Columbia Generations Project (BCGP; *n* = 29,736) linked self-reported chronic disease history data to a Chronic Disease Registry (CDR) that applied algorithms to administrative health data to ascertain diagnoses of multiple chronic diseases in the Province of British Columbia. For the 10 diseases captured by both self-report and the CDR, including asthma, chronic obstructive pulmonary disease (COPD), diabetes, hypertension, multiple sclerosis, myocardial infarction, osteoarthritis, osteoporosis, rheumatoid arthritis, and stroke, we calculated Cohen's kappa coefficient to examine concordance of chronic disease status (i.e., ever/never diagnosed) between the data sources. Using CDR data as the gold standard, we also calculated sensitivity, specificity, and positive-predictive value (PPV) for self-reported chronic disease occurrence. The prevalence of each chronic disease was similar across both data sources. Substantial levels of concordance (0.66–0.73) and moderate to high sensitivities (0.64–0.92), specificities (0.98–0.99) and PPVs (0.55–0.84) were observed for diabetes, hypertension, multiple sclerosis, and myocardial infarction. We did observe degree of concordance to vary by age, sex, body mass index (BMI), health perception, and ethnicity across most of the chronic diseases that were evaluated. While administrative health data are imperfect, they are less likely to suffer from bias, making them a reasonable gold standard. Our results demonstrate that for at least some chronic diseases, self-report may be a reasonable method for case ascertainment. However, characteristics of the study population will likely have impacts on the quality of the data.

## Introduction

By providing accurate and complete data, cancer registries have proven invaluable to efforts aimed at reducing the population burden of cancer, including support of research to identify etiologic factors. Unfortunately, for non-cancer chronic disease, such registries are not available in most jurisdictions. Studies have had to, instead, rely on self-reported information, which may be incomplete (e.g., no information available for deceased subjects), unreliable, suffer from bias, and require considerable resources to obtain (i.e., recontacting cohort study participants). For example, in the British Columbia Generations Project (BCGP), participants were asked to self-report, at time of recruitment, on history of a variety of chronic diseases using a questionnaire (1). Without having participants complete additional follow-up questionnaires, BCGP is unable to support research examining incidence of non-cancer chronic disease.

Administrative health data likely serves as a more complete and objective resource for assessing occurrence of non-cancer chronic disease. Based on Medical Service Plan (MSP) Physician Billing Data, PharmaNet Drug Dispensing History, and Hospital Discharge Abstract Data, the British Columbia (BC) Ministry of Health (MoH) created a Chronic Disease Registry (CDR) using algorithms to identify cases of a variety of non-cancer chronic diseases from as far back as 1992 (2). Recently, as part of a pilot study to develop a system for cross-agency linkage with other data holdings, the BC MoH linked the CDR with a limited BCGP dataset that included self-reported chronic disease status from baseline questionnaires.

The objective of this study was to assess the level of agreement between self-reported history of chronic disease and the CDR for BCGP participants and to explore the impact of various factors on levels of agreement. Results will help inform the comparability of differing sources of information to assess non-cancer chronic disease which is important for designing of epidemiologic studies and planning of follow-up activities.

## Methods

### Study population

This study received approval from the BC Cancer/University of British Columbia Research Ethics Board. BCGP is a prospective cohort study of 29,736 participants aged 35–69 at the time of recruitment, which occurred across BC between 2009 and 2016 (1). At baseline, participants completed a questionnaire ascertaining history of non-cancer chronic diseases. Specifically, participants were asked to report if they were ever diagnosed (yes/no/don't know) with any of the following list of chronic conditions: hypertension, myocardial infraction, stroke, asthma, chronic obstructive pulmonary disease (COPD), major depression, diabetes, liver cirrhosis, chronic hepatitis, Crohn's disease, ulcerative colitis, irritable bowel disease, eczema, lupus, psoriasis, multiple sclerosis, osteoporosis and arthritis (rheumatoid or osteoarthritis). Participants consented to have their data linked with administrative databases.

### Data linkage

A dataset consisting of personal health numbers (PHN), birth year, sex, self-reported chronic disease status, age at time of questionnaire completion, ethnicity, general health perception, and body mass index (BMI; measured at assessment center for 54%, self-reported for 33%, missing for 13%) was created for all 29,736 participants and submitted, using secure file transfer procedures, to the MoH for probabilistic linkage with the CDR. The MoH successfully linked 29,692 of the BCGP participants to the CDR and returned a deidentified dataset with variables indicating diagnosis of chronic diseases for each year between 1992 and 2014. For each chronic disease, an ever/never diagnosis variable was created based on the yearly diagnosis variables. The chronic diseases from the CDR included: ischemic heart disease, heart failure, hypertension, stroke, diabetes, arthritis, chronic kidney disease, mental health conditions, musculoskeletal conditions, neurological diseases, and respiratory diseases. The BCGP medical health history questionnaire captured data for a total of 10 of the chronic diseases from the CDR, including asthma, COPD, diabetes, hypertension, multiple sclerosis, myocardial infarction, osteoarthritis, osteoporosis, rheumatoid arthritis, and stroke. CDR variables for acute myocardial infarction and episodic acute myocardial infarction were combined to create a single variable for myocardial infarction. CDR variables for hemorrhagic stroke, episodic hemorrhagic stroke, ischemic stroke, episodic ischemic stroke, transient ischemic attack, and episodic transient ischemic attack were combined into a single variable for stroke. The CDR variables for asthma, diabetes, and osteoporosis were derived from algorithms that combined hospital discharge codes (ICD-9/10), physician claims codes (ICD-9), and prescription medication drug identification numbers (DIN) (3). The algorithms for COPD, hypertension, multiple sclerosis, and osteoarthritis only used hospital discharge and physician claims data. The algorithms for stroke and myocardial infarction only used hospital discharge data, while the algorithm for rheumatoid arthritis only used physician claims data. Since 36 participants were recruited after 2014 and CDR data were only available up to 2014, these individuals were dropped from the analysis, leaving 29,656 participants in the study.

### Statistical analysis

Analyses of each chronic disease were restricted to those BCGP participants with non-missing questionnaire data for that chronic disease. As such, the total population differed across the disease analyses. For each chronic disease, disease prevalence was assessed using both questionnaire and CDR data. We calculated Cohen's kappa coefficient to examine agreement between the two sources of data. The strength of agreement based on kappa values was interpreted as follows: ≤0 = poor, 0.01–0.20 = slight, 0.21–0.40 = fair, 0.41–0.60 = moderate, 0.61–0.80 = substantial, and 0.81–1 = almost perfect (4). Using CDR data as the gold standard, we also calculated sensitivity, specificity, and positive-predictive value (PPV) for each self-reported chronic disease. Sensitivity corresponded to the fraction of participants with a particular chronic disease who correctly self-reported having that disease. Specificity corresponded to the fraction of participants without a particular chronic disease who correctly self-reported not having that disease. PPV corresponds to the fraction of participants who self-reported having a particular chronic disease that actually had that disease.

Using logistic regression models, odds ratios (OR) with 95% confidence intervals (95% CI) were estimated to evaluate the impact of sex (male, female), ethnicity (white, other), age at time of questionnaire completion (<55, 55–64, ≥65), BMI (18.5– ≤ 24.9, ≥25.0), and general health perception (excellent/very good, good/fair, poor) on concordance between self-report and the CDR (0 = no concordance, 1 = concordance) for each chronic disease. Those participants with unknown BMI or general health perception were excluded from these analyses.

## Results

BCGP participants were predominately female (68.7%), White (81.7%), and <55 years of age (42.0%) at baseline (Table 1). Most participants had BMIs that fell in the “healthy” category (18.5– ≤ 24.9; 39.0%) and rated their own health as very good (41.9%). The number of BCGP participants with non-missing self-reported chronic disease data ranged from 26,543 (osteoarthritis/rheumatoid arthritis) to 28,536 (hypertension) (Table 2). The number of concordant cases of chronic disease ranged from 71 (stroke) to 5,043 (hypertension). The prevalence of each chronic condition was generally similar when comparing self-report and CDR data (Table 2).

**Table 1:** Characteristics of participants (*N* = 29,656).

Characteristics	*N* (%)
Sex	
Male	9,293 (31.3)
Female	20,363 (68.7)
Ethnicity	
White	24,214 (81.7)
Other	5,442 (18.3)
Age at questionnaire (years)	
<55	12,447 (42.0)
55–64	11,697 (39.4)
≥65	5,512 (18.6)
Body mass index	
Healthy (18.5–≤24.9)	11,575 (39.0)
Overweight (≤25.0–≤29.9)	9,091 (30.7)
Obese (≥30.0)	5,038 (17.0)
Unknown	3,952 (13.3)
Health perception	
Excellent	6,070 (20.5)
Very good	12,438 (41.9)
Good	8,278 (27.9)
Fair	1,660 (5.6)
Poor	294 (1.0)
Unknown	916 (3.1)

**Table 2:** Overall measures of agreement between registry and self-reported chronic diseases among participants of the BC generations project.

Chronic disease	N	Concordant cases	Self-report prevalence (%)	CDR prevalence (%)	Kappa	Sensitivity	Specificity	PPV
Asthma	28,319	2,055	12.1	11.1	0.58	0.65	0.95	0.60
COPD	28,380	196	1.6	2.0	0.38	0.35	0.99	0.44
Diabetes	28,535	1,309	6.3	7.1	0.66	0.64	0.98	0.73
Hypertension	28,536	5,043	21.1	24.6	0.71	0.72	0.96	0.84
Multiple Sclerosis	28,489	106	0.6	0.5	0.73	0.82	0.99	0.66
Myocardial Infarction	28,045	231	1.5	0.9	0.69	0.92	0.99	0.55
Osteoarthritis	26,543	1,761	17.4	12.5	0.35	0.53	0.88	0.38
Osteoporosis	28,163	1,078	5.9	7.8	0.53	0.49	0.98	0.65
Rheumatoid Arthritis	26,543	214	2.9	1.7	0.34	0.47	0.98	0.28
Stroke	28,473	71	0.9	0.4	0.36	0.58	0.99	0.27

Kappa statistics comparing self-report to the CDR indicated substantial agreement for diabetes (0.66), hypertension (0.71), multiple sclerosis (0.73), and myocardial infarction (0.69) (Table 2). Moderate agreement was observed for asthma (0.58) and osteoporosis (0.53), and fair agreement was observed for COPD (0.38), osteoarthritis (0.35), rheumatoid arthritis (0.34), and stroke (0.36). When considering the CDR as the gold standard, the sensitivity of self-report was greater than 50% for seven of the ten chronic conditions; the highest sensitivities were observed for hypertension (0.72), multiple sclerosis (0.82), and myocardial infarction (0.92). Specificity of the questionnaire was high across all chronic conditions (>0.85). PPV values were greater than 50% for six of the ten chronic diseases; the highest PPV values were observed for diabetes (0.73), hypertension (0.84), and multiple sclerosis (0.66).

Increasing age was significantly associated with lower levels of concordance between the CDR and self-report for most of the chronic conditions. For example, the odds of concordance for myocardial infarction were significantly reduced among participants aged 55–64 (OR = 0.28; 95% CI: 0.17–0.45) and aged ≥65 years (OR = 0.18; 95% CI: 0.11–0.30) compared to those aged <55 at time of questionnaire completion (Table 3). For asthma, there was some indication of increased levels of concordance among the older age groups, though there was no evidence of a trend with increasing age.

**Table 3:** Association of factors with concordance of registry and self-reported chronic diseases among participants of the BC generations project.

	Odds ratio^a^ 95% CI
	Age (ref. <55)	Sex (Ref. Women)	BMI (Ref. Healthy)	Health perception (Ref. excellent/very good)	Ethnicity (Ref. White)
Chronic condition	55–64	≥65	Men	Overweight	Obese	Good/Fair	Poor	Other
Asthma	1.18	1.12	1.17	0.93	0.80	0.70	0.46	0.99
	1.07–1.31	0.99–1.27	1.06–1.30	0.84–1.03	0.71–0.91	0.63–0.77	0.32–0.68	0.88–1.11
COPD	0.33	0.16	0.95	0.91	0.82	0.38	0.13	0.95
	0.25–0.43	0.12–0.21	0.79–1.14	0.74–1.13	0.65–1.04	0.31–0.46	0.08–0.23	0.75–1.22
Diabetes	0.97	0.75	1.37	0.80	0.46	0.69	0.39	0.76
	0.84–1.12	0.64–0.89	1.18–1.58	0.68–0.94	0.39–0.54	0.60–0.78	0.25–0.64	0.65–0.90
Hypertension	0.65	0.55	0.73	0.67	0.51	0.84	0.74	0.94
	0.59–0.72	0.49–0.61	0.67–0.80	0.61–0.74	0.45–0.57	0.77–0.92	0.51–1.12	0.84–1.06
Multiple sclerosis	0.99	0.79	4.15	1.35	1.26	0.49	0.37	1.69
	0.57–1.76	0.41–1.61	1.92–10.84	0.75–2.51	0.66–2.56	0.29–0.83	0.08–6.64	0.82–4.11
Myocardial infarction	0.28	0.18	0.32	0.77	0.42	0.23	0.06	0.90
	0.17–0.45	0.11–0.30	0.24–0.45	0.50–1.18	0.27–0.63	0.16–0.33	0.03–0.12	0.59–1.38
Osteoarthritis	0.38	0.29	1.77	0.90	0.83	0.66	0.37	1.16
	0.35–0.41	0.26–0.32	1.63–1.93	0.83–0.97	0.75–0.92	0.61–0.71	0.27–0.51	1.04–1.29
Osteoporosis	0.17	0.09	4.62	1.46	1.95	0.67	0.44	0.82
	0.14–0.20	0.08–0.11	3.90–5.52	1.29–1.66	1.66–2.31	0.60–0.76	0.27–0.76	0.71–0.96
Rheumatoid arthritis	0.50	0.33	1.40	0.86	0.68	0.49	0.16	0.80
	0.41–0.60	0.26–0.40	1.18–1.68	0.72–1.04	0.56–0.83	0.42–0.58	0.10–0.25	0.66–0.99
Stroke	0.56	0.31	0.74	0.86	0.78	0.32	0.09	1.09
	0.39–0.79	0.21–0.45	0.56–0.98	0.62–1.21	0.55–1.13	0.24–0.44	0.05–0.19	0.75–1.63

^a^ Odds ratios > 1 indicate increased concordance relative to comparison group; odds ratios < 1 indicate decreased concordance relative to comparison group.

We did observe impacts of sex on levels of concordance across most of the chronic diseases. For diabetes, asthma, multiple sclerosis, osteoarthritis, rheumatoid arthritis, and osteoporosis, levels of concordance were significantly greater for men as compared to women (OR range: 1.17–4.62). For myocardial infarction, stroke, and hypertension, levels of concordance were significantly lower for men compared to women (OR range: 0.32–0.73).

Though not always statistically significant, most of the chronic conditions demonstrated decreased concordance when comparing participants who were overweight and obese to those with a “healthy” BMI (Table 3). There was also evidence of a trend of decreasing concordance with increasing BMI. For example, with diabetes, the ORs for concordance were 0.80 (95% CI: 0.68–0.94) and 0.45 (95% CI: 0.38–0.53) among overweight and obese participants, respectively, as compared to participants with a “healthy” BMI. For osteoporosis, on the other hand, there was significant evidence that the level of concordance increased with increasing BMI.

Compared to those who reported being in excellent/very good health, those participants reporting good/fair health and poor health had significantly reduced levels of concordance across most of the chronic conditions. There was also evidence of a trend of decreasing concordance with decreasing self-reported health status. For example, with myocardial infarction, the ORs for concordance were 0.23 (95% CI: 0.16–0.33) and 0.06 (95% CI: 0.03–0.12) among participants reporting good/fair health and poor health, respectively, as compared to participants reporting excellent/very good health.

For diabetes, rheumatoid arthritis, and osteoporosis, concordance was significantly decreased among those of non-White ethnicities as compared to those of White ethnicity (OR range: 0.76–0.82). For osteoarthritis, concordance was greater among those of non-White ethnicities as compared to those of White ethnicity (OR = 1.16; 95% CI: 1.04–1.28).

## Discussion

Fair to substantial levels of concordance were observed between self-report and the CDR for the 10 chronic diseases evaluated in this study. When considering CDR as the gold standard, specificity of self-report was high across all the chronic diseases. Sensitivity and PPV of self-report were generally more modest across the diseases. Age, sex, BMI, health perception and ethnicity were found to influence the levels of concordance between self-report and CDR for most of the chronic diseases we evaluated.

Concordance between the data sources did not seem to be influenced by disease prevalence. For example, higher kappa statistics were observed for both hypertension (common disease) and multiple sclerosis (uncommon disease). Complexity of the algorithms used to predict chronic disease status also did not seem to influence concordance. For example, in addition to hospitalization and physician visit codes, the algorithm for diabetes considered a list of hundreds of medication codes, while for multiple sclerosis, the algorithm considered only two diagnostic codes (3). However, the questionnaire and CDR demonstrated a high degree of concordance for both diseases. Only algorithms for asthma, diabetes, and osteoporosis included pharmaceutical data. While differences in the ability to recall diagnoses of certain chronic conditions may have contributed to variations in concordance levels, it unlikely explains the low levels of concordance observed for conditions like stroke or COPD.

Our results are largely consistent with previous evaluations of agreement between self-report and administrative health databases on status of various chronic diseases (5–8). For example, an evaluation of Quebec's CARTaGENE cohort (CaG; *n* = 19,996) included eight of the chronic conditions that we evaluated, specifically asthma, COPD, diabetes, myocardial infarction, multiple sclerosis, osteoarthritis, rheumatoid arthritis, and stroke (6). Since both BCGP and CaG are part of the Canadian Partnership for Tomorrow's Health (CanPath), the questionnaires used to assess chronic disease were very similar. However, unlike the CDR, the algorithms used to predict occurrence of asthma and diabetes by CaG did not include data on pharmaceutical drug use.

There tended to be larger discrepancies in the prevalence of the eight diseases between data sources in CaG vs. BCGP. However, levels of concordance between the data sources were generally similar for these chronic diseases between the two cohorts. Like CaG, we found that older age was associated with lower concordance for most of the conditions. We also observed similar impacts of sex across most of the eight chronic diseases. Unlike CaG, we did not have access to a variable indicating levels of health care utilization; heavy usage was found to be associated with reduced concordance across all chronic diseases (6). We did, however, examine self-reported health status which is a predictor of health care utilization (i.e., good health status associated with lower health care utilization) (9), and observed that lower self-reported health status was associated with reduced concordance among most chronic conditions.

There has been limited evaluation of the impacts of BMI and ethnicity on agreement between self-report and administrative health data. The effects of BMI may be tied to poorer health perception among those with higher BMIs. A cross-tabulation of BMI and health perception in our study showed that the proportion of those indicating excellent/very good perceived health significantly decreased with increasing BMI category (*χ*^2^ *p* < 0.0001; results not shown). Contradictory to our findings, a previous study among women found that higher BMI was associated with higher agreement for hypertension but found no impact of BMI on agreement for myocardial infarction, stroke, or diabetes (10). Though we lacked the numbers to look at effects of ethnicity in detail, White vs. non-White ethnicity was only found to impact concordance among four of the chronic conditions. The effects may be related to differences in healthcare utilization (11) or differences in accuracy of self-report tied to health literacy or cultural perceptions of illness (12, 13).

The ability to conduct this evaluation within a large, population-based cohort study is a major strength. However, given the limited scope of the data linkage, we were unable to assess the role of other potentially important factors such as smoking history and socioeconomic status on concordance of chronic disease status. Given the unique socioeconomic characteristics of BCGP, our findings may not directly apply to other populations, so replication in other study populations is needed, particularly evaluation of the impact of various factors, such as age, sex, and BMI on levels of agreement. Another limitation is that while participants were asked to report their lifetime history of chronic disease, the CDR only assessed chronic disease status as far back as 1992. The impact of this truncated assessment timeline on our results is likely to be minimal because participants diagnosed with a chronic disease before 1992 are likely to be still receiving health care for that disease after 1992, which the CDR would have captured.

Though administrative health data is imperfect, it is likely less prone to bias than self-report and can be considered a reasonable gold standard. Results of our analyses, which are consistent with previous studies, suggest that, compared to administrative health data, self-report is a reasonable method for assessing history of certain chronic diseases or, at the very least, excluding the occurrence of certain chronic diseases. However, if researchers decide to rely on self-report to ascertain these conditions, they must carefully consider the potential impacts of characteristics of the study population on the quality of the data.

## Data Availability

The data analyzed in this study is subject to the following licenses/restrictions: Access to self-reported data can be obtained through the BC Generations Project. Access to chronic disease registry data requires permission from the British Columbia Ministry of Health. All inferences, opinions, and conclusions drawn in this manuscript are those of the authors, and do not reflect the opinions or policies of the Data Steward. Requests to access these datasets should be directed to https://www.bcgenerationsproject.ca/researchers/requesting-data-and-biosample-access/.

## References

[1] Dhalla A, McDonald TE, Gallagher R, Spinelli JJ, Brooks-Wilson AR, Lee TK, et al. Cohort profile: the British columbia generations project (BCGP). Int J Epidemiol. (2018) 48:377–8. 10.1093/ije/dyy160 30169793

[2] British Columbia Ministry of Health [creator]. Chronic disease registry. British columbia ministry of health [publisher]. MOH (2022). Available at: http://www.health.gov.bc.ca/data.

[3] Chronic Disease Information Working Group. BC ministry of health. Chronic disease registry – data dictionary (2015). Available at: https://www2.gov.bc.ca/assets/gov/health/conducting-health-research/data-access/chronic-disease-registries-case-definitions.pdf.

[4] Landis JR Koch GG. The measurement of observer agreement for categorical data. Biometrics. (1977) 33:159–74. 10.2307/2529310 843571

[5] Singh JA. Accuracy of veterans affairs databases for diagnoses of chronic diseases. Prev Chronic Dis. (2009) 6:A126. 10.1002/art.20827 19755002 PMC2774640

[6] Payette Y de Moura C Boileau C Bernatsky S Noisel N. Is there an agreement between self-reported medical diagnosis in the CARTaGENE cohort and the Québec administrative health databases? Int J Popul Data Sci (2020) 5:1155. 10.23889/ijpds.v5i1.1155 34232968 PMC7473265

[7] Lix LM, Yogendran MS, Shaw SY, Burchill C, Metge C, Bond R. Population-based data sources for chronic disease surveillance. Chronic Dis Can. (2008) 29:31–8. 10.24095/hpcdp.29.1.04 19036221

[8] Muggah E Graves E Bennett C Manuel DG. Ascertainment of chronic diseases using population health data: a comparison of health administrative data and patient self-report. BMC Public Health. (2013) 13:16. 10.1186/1471-2458-13-16 23302258 PMC3557162

[9] Rosella LC, Kornas K, Yao Z, Manuel DG, Bornbaum C, Fransoo R, et al. Predicting high health care resource utilization in a single-payer public health care system: development and validation of the high resource user population risk tool. Med Care. (2018) 56:e61. 10.1097/MLR.0000000000000837 29189576 PMC6143224

[10] Ho PE, Tan CS, Shawon SR, Eriksson M, Lim LY, Miao H, et al. Comparison of self-reported and register-based hospital medical data on comorbidities in women. Sci. Rep. (2019) 9:3527. 10.1038/s41598-019-40072-0 30837593 PMC6400937

[11] Quen H, Fong A, De Coster C, Wang J, Musto R, Noseworthy TW, et al. Variation in health services utilization among ethnic populations. CMAJ Can Med Assoc J. (2006) 174:787–91. 10.1503/cmaj.050674 16534085 PMC1402387

[12] Hunt SM Bhopal R. Self report in clinical and epidemiological studies with non-English speakers: the challenge of language and culture. J Epidemiol Community Health. (2004) 58:618–22. 10.1136/jech.2003.010074 15194728 PMC1732826

[13] Berkman ND Sheridan SL Donahue KE Halpern DJ Crotty K. Low health literacy and health outcomes: an updated systematic review. Ann Intern Med. (2011) 155:97–107. 10.7326/0003-4819-155-2-201107190-00005 21768583

